# Interlaboratory assays from the fungal PCR Initiative and the Modimucor Study Group to improve qPCR detection of Mucorales DNA in serum: one more step toward standardization

**DOI:** 10.1128/jcm.01525-24

**Published:** 2024-12-31

**Authors:** Steffi Rocchi, Emeline Scherer, P. Lewis White, Audrey Guitton, Alexandre Alanio, Françoise Botterel, Marie Elisabeth Bougnoux, Maria José Buitrago, Massimo Cogliati, Marjorie Cornu, Celine Damiani, Julie Denis, Damien Dupont, Stefan Fuchs, Rebecca Gorton, Pieter-Jan Haas, Ferry Hagen, Rasmus Hare, Xavier Iriart, Corné H. W. Klaassen, Michaela Lackner, Martina Lengerova, Willem J. G. Melchers, Florent Morio, Philippe Poirier, Jan Springer, Stephane Valot, Birgit Willinger, Cristina Mazzi, Mario Cruciani, Rosemary Barnes, J. Peter Donnelly, Jürgen Loeffler, Laurence Millon

**Affiliations:** 1 Chrono-environnement UMR6249, CNRS, University of Franche-Comté 27000 , Besançon, Bourgogne-Franche-Comté, France; 2 Parasitology–Mycology Department, Besançon University Hospital 547597 , Besançon, Bourgogne-Franche-Comté, France; 3 Public Health Wales Mycology Reference Laboratory, University Hospital of Wales 97609 , Cardiff, Wales, United Kingdom; 4 Division of Infection and Immunity, Centre for Trials Research 475118 , Cardiff, Wales, United Kingdom; 5 Laboratoire de parasitologie-mycologie, AP-HP, Hôpital Saint-Louis 55663 , Paris, Île-de-France, France; 6 Mycology Department, Institut Pasteur, Université Paris Cité, National Reference Center for Invasive Mycoses and Antifungals, Translational Mycology research group 555089 , Paris, Île-de-France, France; 7 Unité de Parasitologie-Mycologie, Département de Prévention, Diagnostic et Traitement des Infections, CHU Henri Mondor, Assistance Publique des Hôpitaux de Paris (APHP), Créteil, France; 8 Parasitology-Mycology Unit, Necker Enfants Malades Hospital, APHP 246596 , Paris, Île-de-France, France; 9 Mycology Reference Laboratory, Centro Nacional de Microbiologia, Instituto de Salud Carlos III 38176 , Madrid, Community of Madrid, Spain; 10 CIBERINFEC, ISCIII-CIBER de Enfermedades Infecciosas, Instituto de Salud Carlos III 38176 , Madrid, Community of Madrid, Spain; 11 Department of Biomedical Sciences for Health, Università degli Studi di Milano 9304 , Milan, Italy; 12 Université de Lille, Inserm U1285, CHU Lille, Laboratoire de Parasitologie-Mycologie, CNRS, UMR 8576, UGSF—Unité de Glycobiologie Structurale et Fonctionnelle 27023 , Lille, Hauts-de-France, France; 13 Laboratoire de Parasitologie et Mycologie Médicales, Centre de Biologie Humaine, CHU Amiens Picardie, Agents Infectieux, Résistance et Chimiothérapie (AGIR), UR 4294, Université de Picardie Jules Verne 26993 , Amiens, Hauts-de-France, France; 14 Laboratoire de Parasitologie et de Mycologie Médicale, Hôpitaux Universitaires de Strasbourg 36604 , Strasbourg, Grand Est, France; 15 Institut des Agents Infectieux, Service de Parasitologie et Mycologie Médicale, Hospices Civils de Lyon, Hôpital Croix-Rousse 423788 , Lyon, Auvergne-Rhône-Alpes, France; 16 Molecular Diagnostics, Institute of Hygiene and Medical Microbiology, Medical University of Innsbruck 27280 , Innsbruck, Tyrol, Austria; 17 Health Services Laboratories, London, United Kingdom; 18 Department of Medical Microbiology, University Medical Center Utrecht, Utrecht, the Netherlands; 19 Westerdijk Fungal Biodiversity Institute 141042 , Utrecht, the Netherlands; 20 Mycology Unit, Department for Bacteria, Parasites and Fungi, Statens Serum Institut 4326 , Copenhagen, Capital Region of Denmark, Denmark; 21 Service de Parasitologie-Mycologie, CHU Toulouse 36760 , Toulouse, France; 22 Institut Toulousain des Maladies Infectieuses et Inflammatoires (Infinity), Université de Toulouse, CNRS UMR5051, INSERM UMR1291, Universite Paul Sabatier 27091 , Toulouse, Occitanie, France; 23 Department of Medical Microbiology and Infectious Diseases, Erasmus MC University Medical Center Rotterdam 6993 , Rotterdam, South Holland, The Netherlands; 24 Mycology Research Group, Institute for Hygiene and Medical Microbiology, Medical University of Innsbruck (MUI) 27280 , Innsbruck, Tyrol, Austria; 25 Department of Internal Medicine—Hematology and Oncology, University Hospital Brno 48243 , Brno, South Moravian Region, Czechia; 26 Department of Medical Microbiology, Radboud University Medical Center 6034 , Nijmegen, Gelderland, the Netherlands; 27 Nantes Université, CHU Nantes, Cibles et Médicaments des Infections et de l'Immunité 26922 , Nantes, France; 28 Laboratoire de Parasitologie-Mycologie, CHU Clermont-Ferrand, 3IHP, Paris, France; 29 Department of Internal Medicine II, WÜ4i, University Hospital Wuerzburg, Wuerzburg, Germany; 30 Laboratoire de Parasitologie-Mycologie, Plateforme de Biologie Hospitalo-Universitaire Gérard Mack, Dijon, France; 31 Division of Clinical Microbiology, Department of Laboratory Medicine, Medical University of Vienna 27271 , Vienna, Austria; 32 IRCCS Sacro Cuore Don Calabria Hospital, Negrar di Valpolicella, Verona, Italy; 33 Fungal PCR Initiative (FPCRI), Verona, Italy; 34 Medical Microbiology and Infectious Diseases, Cardiff University School of Medicine 2111 , Cardiff, United Kingdom; 35 Fungal PCR Initiative (FPCRI), Nijmegen, the Netherlands; University of Utah, Salt Lake City, Utah, USA

**Keywords:** mucormycosis, Mucorales PCR, standardization, interlaboratory assay

## Abstract

The aim of this study was to identify parameters influencing DNA extraction and PCR amplification efficiencies in an attempt to standardize Mucorales qPCR. The Fungal PCR Initiative Mucorales Laboratory Working Group distributed two panels of simulated samples to 26 laboratories: Panel A (six sera spiked with Mucorales DNA and one negative control serum) and Panel B (six Mucorales DNA extracts). Panel A underwent DNA extraction in each laboratory according to the local procedure and were sent to a central laboratory for testing using three different qPCR techniques: one in-house qPCR assay and two commercial assays (MucorGenius and Fungiplex). Panel B DNA extracts were PCR amplified in each laboratory using local procedures: nine in-house qPCR assays and two commercial kits (MucorGenius and MycoGENIE). All data were compiled and anonymously analyzed at the central laboratory. For Panel A, a total of six different automated platforms and five manual extraction methods were used. Positive rates were 64%, 70%, and 89%, for the MucorGenius, Fungiplex, and the in-house qPCR assay, respectively. Using a large volume of serum for DNA extraction provided the highest analytical sensitivity (82.5% for 1 mL compared with 62.7% for smaller volumes, *P* < 0.01). For Panel B, five in-house qPCR assays and two commercial kits had >78% positivity. Using larger PCR input volumes (≥7 µL) was associated with the highest sensitivity at 95.5% compared to 58.3% when lower input volumes were used (*P* < 0.01). Using larger sample volumes for nucleic acid extraction and DNA template volumes for PCR amplification significantly improves the performance of Mucorales qPCR when testing serum.

**IMPORTANCE:**

Mucormycosis is a life-threatening mold infection affecting immunosuppressed patients but also other patients with diabetes or trauma. Better survival is linked to shorter delays in diagnosis and treatment initiation. Detection of Mucorales-free DNA in serum or plasma using quantitative PCR allows a prompt diagnosis and earlier treatment. Several techniques and protocols of quantitative Mucorales PCR are used in Europe, and improving performance remains a common objective of laboratories participating in the fungal PCR Initiative Working Group. This study, which combined results from 26 laboratories in Europe, showed that the main parameters underpinning sensitivity are the preanalytical variables (volume of serum used for DNA extraction and DNA template volume), irrespective of the extraction platforms and qPCR assay/platform.

## INTRODUCTION

Mucormycosis remains a difficult-to-diagnose, life-threatening disease caused by fungi from the order Mucorales. Clinical and radiological signs are not specific and can be confused with invasive aspergillosis, a more common invasive mold infection. However, early differentiation between mucormycosis and invasive aspergillosis is essential, given the rapid progression of infection and the requirement for different treatments. Early treatment of mucormycosis includes systemic antifungal (e.g., lipid-based amphotericin B formulations as first-line agents) and, whenever possible, surgery to improve survival in immunosuppressed patients (1). In recent years, the development of qPCR assays to detect Mucorales DNA in blood samples has markedly improved the diagnosis of mucormycosis, allowing earlier appropriate therapy.

Whole blood analysis potentially enables multiple DNA sources (e.g., intracellular DNA, free DNA, and cell-associated DNA) to be targeted at the same time but current protocols typically do not target-free DNA. When targeting DNA associated with fungal cells (whether phagocytosed or not), centrifugation of whole blood deposits fungal cells together with the blood cells, and DNA can be subsequently extracted from the pellet using complex and time-consuming extraction protocols. In contrast, free DNA (DNAemia), which consists of fragments of fungal DNA, is likely the only source of DNA in plasma/serum with the fungal cells likely lost during the fractionation of blood. Circulating free DNA can be extracted using a simple extraction protocol, avoiding the critical issue of fungal cell wall lysis. The high sensitivity of cell-free DNA detection in serum or plasma has been hypothesized to be due to the potential extensive angioinvasion of Mucorales along with a high copy number of rRNA and the lack of hyphal cross-walls that cannot prevent the release of extensive cellular contents when hyphae are damaged (2, 3). Several clinical studies showed that the sensitivity of the Mucorales qPCR on serum ranges from 80% to 90%, and positivity precedes histological/mycological evidence by approximately 4 days, and radiological signs by 1 day (2, 4–6). Finally, longitudinal fungal cell-free DNA testing provides a measure of the fungal burden through the quantification cycle (Cq) value and can be used to predict outcomes (7, 8).

Despite the recent availability of commercial kits and an increasing number of studies showing sufficient performance for the diagnosis of mucormycosis, Mucorales qPCR was not included as a mycological criterion for probable mucormycosis in recent revisions of the European Organization for Research and Treatment of Cancer/MycosesStudy Group Education and Research Consortium (EORTC/MSGERC) definitions (9–13). Similarly, to *Aspergillus* PCR, there was reluctance to include this criterion based on the perceived lack of standardization.

With the aim of standardizing Mucorales PCR, the first interlaboratory evaluation of Mucorales qPCR assays was organized in 2017–2018 by the ISHAM Working Group, the Fungal PCR Initiative (FPCRI). Twenty-one laboratories within Europe participated in this first FPCRI Mucorales PCR evaluation (14). Despite the variety of techniques used, a very low interlaboratory variability in Cq values was observed (standard deviation = 1.89 cycles). However, with 26 different protocols described, there were too many different combinations of DNA extraction and amplification techniques to be able to identify key parameters that may have affected the performance of Mucorales PCR.

Irrespective of the test (commercial or in-house kit), analytical validation using contrived but clinically relevant samples is essential to identify key parameters allowing performance optimization, particularly of essential procedures beyond PCR amplification itself (e.g., nucleic acid extraction). This strategy must be performed before clinical validation using patient samples making it possible to identify optimal options without sacrificing precious samples.

The aim of the current interlaboratory exercise was to individually assess the DNA extraction and the amplification steps using samples spiked with fungal DNA, to identify key parameters that influence qPCR performance. The FPCRI Mucorales-Laboratory Working Party (M-LWP) organized the distribution of two simulated sample panels: one panel of sera spiked with Mucorales DNA to assess the nucleic acid extraction protocols and one panel of Mucorales DNA extracts to assess PCR amplification protocols.

## MATERIALS AND METHODS

### Participants

Twenty-six laboratories participated in the two trials, Panel A assessing DNA extraction and Panel B assessing qPCR amplification. The participants comprised 12 laboratories from French university hospitals, nine of them having participated in the French national prospective Modimucor study (8), and 14 laboratories participating in the M-LWP, from eight European countries. Results were anonymized for analysis. Post-analysis and for external quality assessment purposes, each participant received their individual results, compared with the overall data from other laboratories.

### Preparation of simulated samples

The overall approach was to perform an external quality control study using serum spiked with fungal DNA to assess nucleic acid extraction and PCR amplification of cell-free DNA. Serum for Panel A was obtained from one healthy donor, and serum for Panel B was taken from another individual, with blood donated specifically for research purposes, following the procedures and ethical rules of the Bourgogne Franche-Comté Blood Transfusion Centre.

Three strains belonging t*o Rhizomucor pusillus* (Centre de Ressources Biologiques—Filière Microbiologique, Besançon [CRB-FMB], Biobanque BB-0033-00090), *Rhizopus arrhizus* (CBS 329.47), and *Lichtheimia corymbifera* (IHEM 3809), all grown on Sabouraud dextrose agar medium (37°C, 5 days), were used to prepare the simulated samples. These three species were chosen because they are representative of the main agents causing mucormycosis in Europe (1, 15). The DNA concentrations chosen generated Cq values comparable to those observed in patients diagnosed with mucormycosis (range 23–41 cycles) (2). For each species, the genome equivalents were assessed by extracting DNA from a suspension of quantified spores serially diluted to reach the desired concentrations, assuming that a conidium contains a single genome and that DNA extraction efficiency is 100% (16). DNA was extracted using the DNeasy Plant Mini Kit (Qiagen, Hilden, Germany) as previously described (14). Primary DNA solutions (DNA-1) were stored at −20°C before being used to prepare Panel A (simulated DNA-spiked sera) and Panel B (DNA extract samples).

#### Simulated serums (Panel A)

Panel A consisted of one negative control serum and six 1 mL simulated sera spiked using the DNA-1 solutions. Two concentrations (5 and 50 genome equivalents/mL) were prepared for each species (*Rhizomucor pusillus*, *Rhizopus arrhizus*, and *Lichtheimia corymbifera*) at the central laboratory (Mycology Unit, Besançon University Hospital, France).

Serum samples from Panel A were stored at −20°C and then sent on dry ice to the 26 laboratories. The seven 1 mL serum samples underwent DNA extraction in each laboratory according to local procedures. All DNA extracts (seven per laboratory) were sent back to the central laboratory on dry ice.

#### DNA extracts (Panel B)

For each Mucorales species, 8 mL of serum was spiked with the DNA-1 solution to reach a concentration of 5,000 genome equivalents/mL. Then, 8 × 1 mL spiked serum was extracted at the central laboratory using Magna Pure Compact extraction platform, providing eight extracts of 50 µL for each species, with a theoretical concentration of 5,000 genome equivalents/mL (assuming 100% DNA extraction efficiency). The eight extracts were pooled to provide 400 µL of a secondary DNA solution (DNA-2) for each species. The DNA-2 solution was diluted in Tris EDTA (TE) buffer to provide concentrations of 5 and 50 genome equivalents/mL for *R. pusillus* and *L. corymbifera,* similar to our previous interlaboratory assay (14). For *R. arrhizus*, as we had no experience of the possibilities of detecting low quantities by other qPCR, in other laboratories, samples with higher concentrations (50 and 500 genome equivalents/mL) were sent. Each diluted DNA-2 solution was divided to provide aliquots of 50µL for each fungal species at each concentration.

Panel B therefore consisted of six 50 µL DNA samples, which were stored at −20°C and sent frozen to the 26 laboratories, along with Panel A. The six DNA samples were amplified in each laboratory according to the local procedures. A negative control sample was not included in Panel B, given each center would be expected to run a no-template control when performing PCR locally.

### DNA extraction and qPCR assays (interlaboratory assay/Panel A and Panel B)

Each participating laboratory was asked to provide detailed technical information regarding the local procedures used for nucleic acid extraction for Panel A (including volume of serum extracted, elution volume, and extraction platform) and PCR amplification of Panel B (volume of DNA template [DNA input], final volume of qPCR, PCR template percentage [defined as the ratio of the DNA template volume divided by final volume of qPCR × 100], qPCR platform and mastermix, qPCR assay used) through an on-line technical form (Fig. S1).

The DNA extraction techniques used for Panel A are shown in Table S1. The 182 DNA extracts (seven DNA extracts from 26 participants) returned frozen to the central laboratory were analyzed using a previously described in-house Mucorales qPCR (IH1) (2) and the commercial Mucorales detection kits MucorGenius and Fungiplex (see details in supplementary data and Tables S2 and S3). All the DNA extracts were thawed on the same day and stored at 4°C, and all the qPCR amplifications were done within 1 week in the same facility (molecular biology platform, UMR Chrono-environnement, Besançon, France) on the same qPCR platform (QuantStudio 5, ThermoFisher Scientific), and by the same laboratory technician.

Technical details of qPCR assays for Panel B-DNA are presented in Table S4.

### Statistical analyses

For analytical purposes, negative results were allocated a value of 45 cycles. Statistical analyses were performed using the statistical software R-3.4.4 for Microsoft Windows.

For Panel A, three full linear mixed models (LMMs)(17) with a “sample” variable in a random part of the model were undertaken to model Cq values obtained for the three qPCR assays as a function of volume of serum sample used for DNA extraction and elution volume. When both variables (volume of sample extracted and elution volume) were statistically significant in the first LMM, a ratio for DNA extraction (elution volume/volume of sample extracted × 100) was used for the second LMM to compare the DNA extraction method. When this was not the case, only the volume (sample or elution) was kept in the second LMM to compare DNA extraction method. For each LMM (used to analyze data of Panels A and B), inclusion of the “sample” variable as a random part was tested and improved the model every time. Backward stepwise selections were performed for the different LMM to select variables to include in the final model.

Then, positivity rates for each level of sample volume or elution volume were determined, and analytical sensitivities were calculated. Differences in analytical sensitivity were assessed by Fisher’s exact test, and sample volume or elution volume associated with improved sensitivity were defined.

For Panel B, a first LMM with a “sample” variable in the random part of the model was undertaken to model Cq values obtained as a function of the qPCR assay used by centers, and qPCR assays were pairwise compared with lmerTest library. Technical parameters that influenced performance (reagent mix, qPCR platform, qPCR input volume, final volume of qPCR reaction) were assessed using LMM in two ways: (i) for qPCR assays for qPCR which showed no difference in performance with “sample” and qPCR “assay” variable in random part of the model and (ii) only for IH1, which was used by 13 participants, to eliminate potential variability due to the qPCR assay. Sensitivities were then calculated as previously explained for the different categories of volumes (input DNA or template percentage) and compared with Fisher’s exact test.

## RESULTS

### Parameters of DNA extraction that influence qPCR results (Panel A)

DNA extracted at the 26 participating laboratories was analyzed using three assays (IH1, Fungiplex, and MucorGenius) at the central laboratory. To provide confidence in the accuracy of qPCR results, data from two centers were excluded from the analysis due to either potential *Mucor* spp. contamination during the extraction step or PCR inhibition which could have undermined PCR positivity or negativity, respectively. Positivity rates were 64%, 70%, and 89%, for the MucorGenius, Fungiplex, and the in-house qPCR assay, respectively (Table 1). A total of six different automated platforms and associated nucleic acid extraction kits were used by 21 laboratories: (i) Roche MP24 (Roche Diagnostics, Mannheim, Germany) (*n* = 7); (ii) NucliSENS easyMAG (bioMérieux, Marcy-l’Étoile, France) (*n* = 6); (iii) QIAsymphony + EZ1 (Qiagen) (*n* = 3); (iv) Ingenius (ELITech Group, Spankeren, Belgium) (*n* = 3); (v) Starlet (Hamilton, Reno, NV, USA) (*n* = 1); (vi) MT-PREP (AusDiagnostics, Chesham, United Kingdom) (*n* = 1). The remaining five laboratories used manual extraction methods with four different manual kits (Table S1). Most (58%) laboratories used 1 mL of serum for extraction (*n* = 15), the others used less than 0.5 mL (0.2 mL [*n* = 6], 0.3 mL [*n* = 1], 0.4 mL [*n* = 2], or 0.5 mL [*n* = 2]). The elution volume was mainly 50 µL (*n* = 15), although huge variations were observed (50–165 µL) (Table S1).

**TABLE 1:** Analytical performance of three qPCR assays for the detection of Mucorales DNA in serum (Panel A)^*a*^^,^^*b*^

	Positivity rates (% [*n*/*N*, 95% CI])		
Sera and composition	IH1	Fungiplex	MucorGenius
S1—*Rhizomucor pusillus* (5 genome eq/mL)	63 (15/24, 43–79)	8 (2/24, 2–26)	13 (3/24, 4–31)
S2—*Rhizomucor pusillus* (50 genome eq/mL)	88 (21/24, 69–96)	54 (13/24, 35–72)	79 (19/24, 60–91)
S3—*Lichtheimia corymbifera* (5 genome eq/mL)	92 (22/24, 74–98)	88 (21/24, 69–96)	46 (11/24, 28–65)
S4—*Lichtheimia corymbifera* (50 genome eq/mL)	100 (24/24, 86–100)	96 (23/24, 80–100)	79 (19/24, 60–91)
S5—*Rhizopus arrhizus* (5 genome eq/mL)	92 (22/24, 74–98)	79 (19/24, 60–91)	67 (16/24, 47–82)
S6—*Rhizopus arrhizus* (50 genome eq/mL)	100 (24/24, 86–100)	96 (23/24, 80–100)	100 (24/24, 86–100)
All samples	89 (128/144, 83–93)	70 (101/144, 62–77)	64 (92/144, 56–71)

	Cq values		
Distribution of Cq values (positive qPCR)	IH1	Fungiplex	MucorGenius
Minimum Cq	25.95	29.75	25.02
Median Cq	31.55	33.65	31.00
Mean Cq	31.64	34.26	31.24
Maximum Cq	39.74	43.06	43.52

^*a*^ IH1: in-house qPCR assay (2).

^*b*^ DNA extracts were returned from 26 centers but data analysis was performed on 24 data sets due to potential contamination or inhibition that would have unduly influenced analysis.

**Fig 1:**
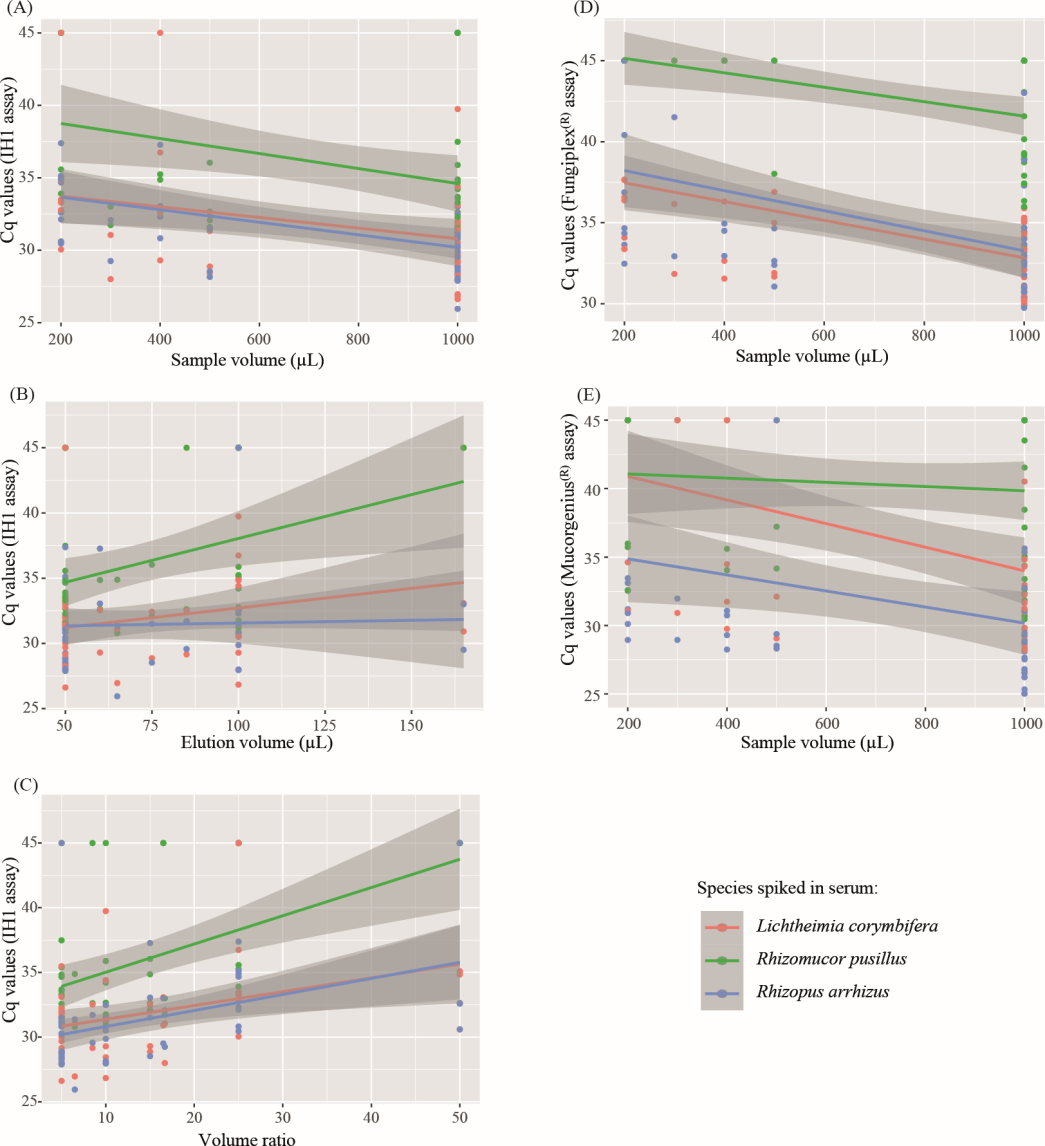
Variability of Cq values according to volumes: (**A**) IH1 Cq and sample volume used for DNA extraction, (**B**) IH1 Cq and elution volume, (**C**) IH1 Cq and volume ratio = elution volume/sample volume extracted × 100, (**D**) Fungiplex Cq and sample volume used for DNA extraction, (**E**) MucorGenius Cq and sample volume used for DNA extraction. IH1: in-house qPCR assay (2). Negative results were allocated a value of 45 cycles.

For the IH1 qPCR assay, the volume of serum sample used for DNA extraction and elution volume were significantly associated with improved performance in the first LMM (*P* < 0.01 for both). In the second LMM, including DNA automated extraction method, only the volume ratio variable (elution volume/volume of sample extracted) was significant for inclusion in the model (*P* < 0.01). For Fungiplex and MucorGenius assays, only the initial sample volume was significant in both LMMs (*P* < 0.01). No difference in Cq was observed between the different types of automated DNA extraction methods. Relationships between Cq values and volumes used for DNA extraction (volume of serum used for extraction, elution volume, and ratio) are presented in Fig. 1. For all qPCR assays, improved qPCR performance correlated with a larger sample volume.

Sensitivities of all qPCR assays (for the 24 centers) were finally compared for large sample volumes (1 mL) vs lower sample volumes and for low elution volumes (50 µL) vs larger elution volumes (Table 2). Improved analytical sensitivity was associated with using a larger sample volume (82.5% for 1 mL sample and 62.7% for other volumes, *P* < 0.01). Sensitivity results according to volume parameters for IH1, Fungiplex, and MucorGenius are provided in Table S5.

**TABLE 2:** qPCR assay sensitivity according to volumes parameters for DNA extraction and DNA amplification^*a*^^,^^*b*^

	Volumes	Sensitivity (%)	Fisher’s exact test
Panel A DNA extraction	Sample volume = 1 mL	82.5	<0.01
	Sample volume < 1 mL	62.7	
	Elution volume = 50 µL	77.4	0.18
	Elution volume > 50 µL	71.7	
Panel B qPCR reaction	DNA template volume ≥ 7 µL	95.8	0.01
	DNA template volume < 7 µL	81.6	
	Template percentage^*c*^ ≥ 35	95.8	0.01
	Template percentage^*c*^ < 35	81.6	

^*a*^ Panel A: serum and elution volumes used in the DNA extraction step (24 centers, 144 qPCR trials by qPCR assays) (two centers excluded from the analysis due to contamination or PCR inhibition).

^*b*^ Panel B: template and final volume used in qPCR reactions (22 centers, 132 qPCR trials) (four centers excluded from the analysis due to lower positivity rates).

^*c*^ Template percentage = (template volume/final volume) x 100.

There was a trend for improved analytical sensitivity with the use of lower elution volumes (77.4% for 50 µL and 71.7% for higher volumes, *P* = 0.18).

### Parameters that influenced DNA amplification (Panel B)

Overall, 26 data sets were returned. Nineteen of the participating laboratories used six previously published in-house qPCR assays: IH1 (*n* = 13) (2), IH2 (*n* = 2) (18), IH3 (*n* = 1) (6), IH4 (*n* = 1) (19), IH5 (*n* = 1) (20), IH6 (*n* = 1) (21). Three laboratories used unpublished in-house qPCR assay. Three laboratories used the MucorGenius (PathoNostics), and one laboratory used MycoGENIE (Ademtech, Pessac, France). Final volume of the PCR mix was mainly 20 µL (*n* = 12) or 25 µL (*n* = 10), and occasionally 10, 15, 30, or 50 µL (once, each). Input DNA volume was mainly 9 µL (*n* = 9) or 5 µL (*n* = 9), and occasionally 6–8 µL (*n* = 4). Only two laboratories used an input DNA volume ≤2 µL (Table S4).

**Fig 2:**
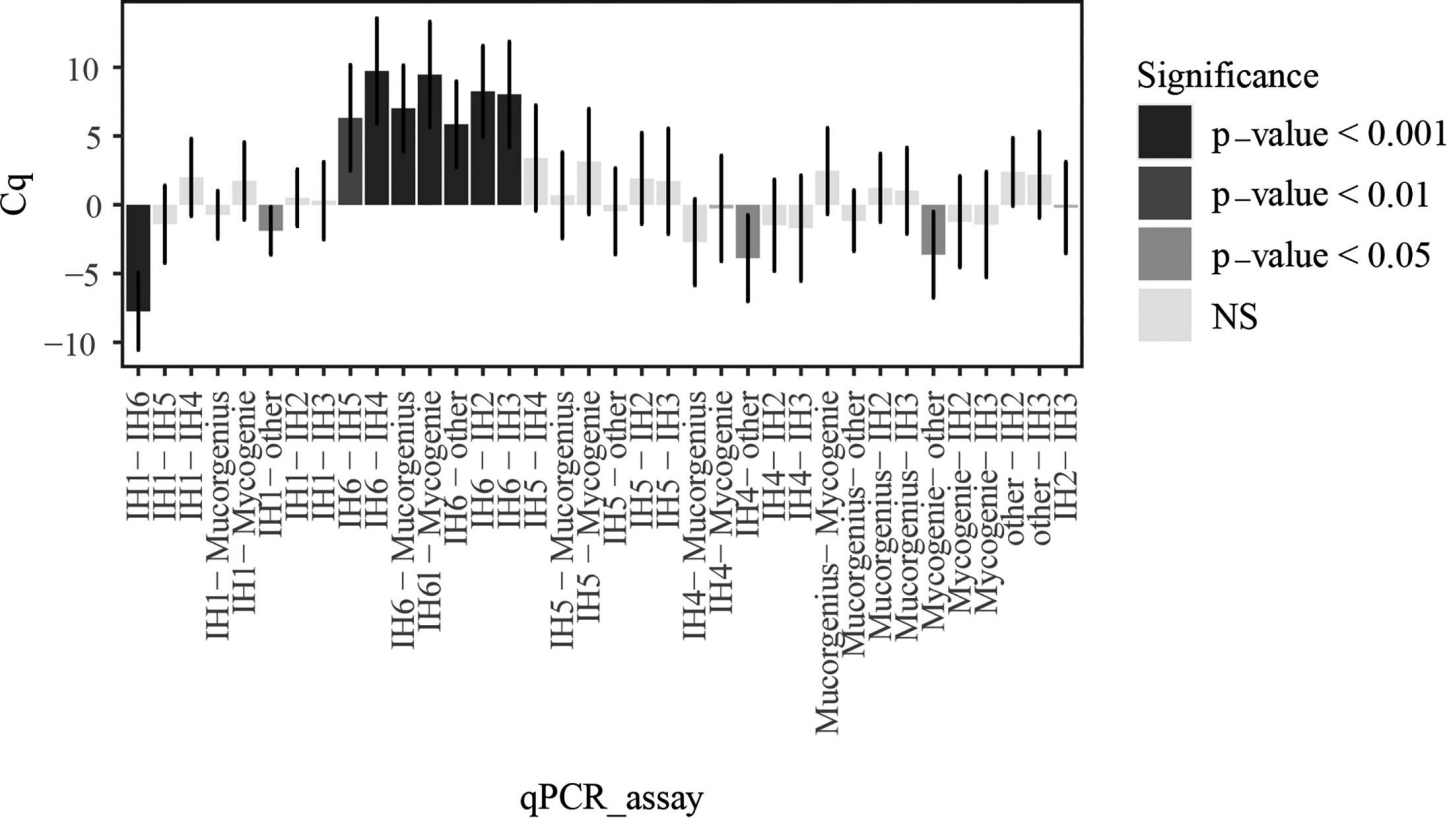
Pairwise comparison of the quantification cycle (Cq) values generated by the qPCR assays (least squares means and confidence intervals between the qPCR assay included in the fixed part of linear mixed effects model). Significance of differences is mentioned with gray intensities. When the difference had a negative value, the first qPCR assay had a best performance and vice versa. NS: no significant difference. Negative results were allocated a value of 45 cycles. IH: in-house qPCR assays described in papers as follows: IH1: ref. (2), IH2: ref. (18), IH3: ref. (6), IH4: ref. (19), IH5: ref. (20), IH6: ref. (21). Other: in-house unpublished method.

Positivity rates varied from 28% to 100% depending on the qPCR assays (Table 3). LMM aiming to compare qPCR performances showed significant differences between qPCR assays. Pairwise comparisons of Cq values generated by each assay (Fig. 2) showed that IH6 qPCR assay showed poor performances compared with all qPCR assays (*P* < 0.01), and “other” qPCR assays (in-house unpublished methods) showed poor performances compared with the IH1, IH4, and MycoGENIE assays (*P* = 0.03, 0.01, and 0.02, respectively). As IH6 and “others” had significantly lower positivity rates compared to the remaining qPCR assays (Table 3, 33% and 28%, respectively), they were removed prior to further analysis to avoid potential bias, as it was felt that their inferior performance was associated with the individual qPCR assay design rather than specific technical aspects of the process. IH6 qPCR was performed on LC480 II (Roche), a platform also used by four other centers, which obtained good results (with other qPCR assays). “Other” qPCR assays were performed on several platforms (InGenius [ELitech], CFX96 [Bio-Rad], and LC480 II) also used by other centers that had achieved good results with other types of qPCR assay.

**TABLE 3:** Proportion of DNA samples detected positive according to each qPCR assay for Panel B testing^*a*^

	Number of centers	Total number of qPCR reactions^*b*^	Number of positive results	Percentage of positive results (95% CI)
IH1	13	78	70	90 (81–95)
IH2	2	12	12	100 (76–100)
IH3	1	6	6	100 (61–100)
IH4	1	6	5	83 (44–97)
IH5	1	6	5	83 (44–97
IH6	1	6	2	33 (10–70)
MycoGENIE	1	6	6	100 (61–100)
MucorGenius	3	18	14	78 (55–91)
Others	3	18	5	28 (13–51)

^*a*^ IH: in-house qPCR assays described in papers as follows: IH1: (2), IH2: (18), IH3: (6), IH4: (19), IH5: (20), IH6: (21). Others: in-house unpublished method.

^*b*^ Number of centers performing the specific assay multiplied by the total number of samples in Panel B (*n* = 6).

**Fig 3:**
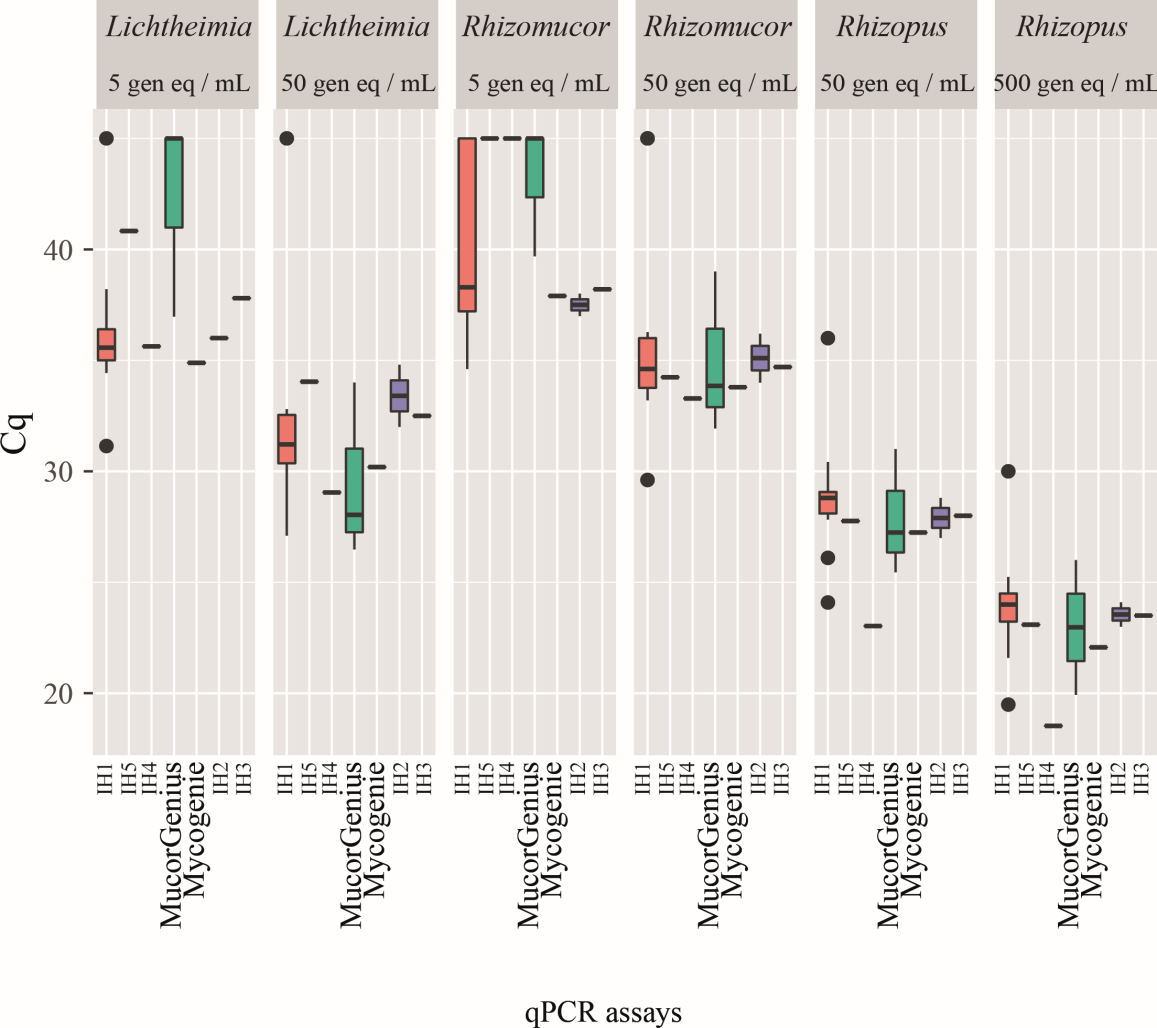
Distribution of Cq values according qPCR assays (*n* = 22 centers, 132 qPCR trials [four centers excluded from the analysis due to lower positivity rates]). IH: in-house qPCR assays described in papers as follows: IH1: ref. (2), IH2: ref. (18), IH3: ref. (6), IH4: ref. (19), IH5: ref. (20). Negative results were allocated a value of 45 cycles.

Distribution of Cq values according to qPCR assays is presented in Fig. 3. Only the DNA template volume or template percentage was significant for inclusion in the LMM (*P* < 0.01). The type of qPCR platform (*n* = 11) did not influence the qPCR performance in this analysis. For qPCR platform used by laboratories included in the analysis, LC480, LC480 II, or LightCycler 2.0 were used by eight laboratories, QuantStudio 5 or Applied 7500 was used by six laboratories, Step One plus by two laboratories, RotorGene Q or 6000 by three laboratories, CFX96, InGenius or MIC by one laboratory each (Table S4).

Technical parameters were also studied solely for the IH1, given 13/26 participating centers (including the central laboratory) used this test with 90% of results (70/78) being positive. DNA input volume and final volume, and subsequently the template percentage, were significant for inclusion in the LMM (*P* < 0.01). The type of qPCR platform (*n* = 7) did not influence the qPCR performance in this analysis. Sensitivities of qPCR according to DNA template and template percentage are presented in Table 2. Using larger DNA input volumes (≥7 µL) was associated with improved sensitivity at 95.8% compared to 81.6% when using lower volume (*P* = 0.01). A template percentage ≥35 (corresponding to 7 µL of DNA template in 20 µL final) was associated with improved sensitivity at 95.8% compared to 81.6% for a lower percentage (*P* = 0.01).

## DISCUSSION

Based on the previous interlaboratory study, we hypothesized that optimal qPCR performance depends on a combination of technical parameters (14). In this current study, the initial sample volume used for DNA extraction and the volume of DNA input used for qPCR are the two most important parameters governing the performance of Mucorales PCR. Although these results are relatively intuitive in relation to optimal analytical sensitivity, they remain pertinent in routine practice, as specimens and DNA extracts are regularly used for the detection of different pathogens across molecular platforms.

Technical optimization for concentrating the DNA in the specimen submitted to qPCR assay is a key step and should be performed before the clinical validation stage, for both in-house and commercial tests to avoid misleading clinical performance data and the subsequent waste of critical samples. Overall, the qPCR detection of Mucorales DNA in spiked serum samples was satisfactory, with majority of the participating laboratories detecting serum samples containing small concentrations of DNA (five genomes equivalent/mL of serum). Although complementary studies done with real clinical samples are needed, this result is very encouraging, showing that several DNA extraction techniques and qPCR assays produce consistent performance. This consistency allows reliable Mucorales qPCR results to be generated in centers equipped with ranging molecular biology platforms and does not restrict the technique to expert laboratories. This technique could therefore enhance the diagnosis of mucormycosis in many clinical settings, and this study provides additional evidence for the potential inclusion of Mucorales PCR in future EORTC/MSGERC definitions.

When assessing extraction parameters (Panel A), similar qPCR performances were observed irrespective of the automatic extractor used. The most critical factor was the volume of serum used for DNA extraction. We demonstrated an increased sensitivity to 82.9% for 1 mL sample compared to 62.7% for lower sample volumes. It is therefore essential to perform DNA extraction on the largest possible volume of serum. Elution volume can also modify qPCR performance, with an increase in sensitivity by reducing the elution volume (and consequently an increased DNA concentration in the eluate), especially marked for IH1 qPCR when the elution volume is equal to 50 µL.

Contrived samples for this interlaboratory assay were prepared using serum for practical reasons (i.e., easy and low-cost supply of matrix), and they also represent an easy sample to process in the molecular diagnostic laboratory, and clinical performance has been demonstrated (8). Of note, a previous study from the European *Aspergillus* PCR Initiative has demonstrated better sensitivity of *Aspergillus* PCR when performed on plasma compared with serum, due to loss of trapped cell-free DNA during clot formation (22, 23). Better performance of fungal cell-free plasma DNA detection was also demonstrated in patients with invasive and non-invasive fungal infections based on preanalytical optimization studies (7, 24). Sensitivity of *Aspergillus* PCR was increased as much as 93% by performing cell-free DNA extraction using a 4 mL plasma volume (25). Plasma cell-free DNA sequencing for diagnosing invasive mold infection also seems very promising (26). While recent articles recommend the use of 4 mL plasma volume for DNA fungal extraction to increase sensitivity, its routine application in the clinical setting will be limited by suitable extraction platform availability. Mucorales qPCR tends to frequently take place very early in the management of invasive mold infections, with both *Aspergillus* and Mucorales PCR performed simultaneously in immunosuppressed patients. Using 1 mL of plasma or serum sample for DNA extraction helps to improve performance, while being feasible in the context of a twice-a-week screening of high-risk patients and increases the number of potential extraction platforms that can be utilized.

Using the second panel (Panel B), we were able to demonstrate that seven distinct qPCR assays had optimal performances, the exception being IH6 and a group of “others” (corresponding to unpublished in-house assays). We have therefore chosen to remove the data from centers using IH6 or other qPCR assays from the following analyses. When assessing the influence of technical parameters on qPCR performance for the optimal qPCR assays, DNA template volume and template percentage were the only significant parameters impacting sensitivity, with the larger DNA template volume particularly when compared to the final volume, resulting in better qPCR performance. However, we have not tested Panel B in the central laboratory with the three qPCRs (IH1, MucorGenius, and Fungiplex, like Panel A), which could have provided additional information.

A limitation of our approach of spiking genomic DNA into serum to assess molecular techniques targeting circulating cell-free DNA is the likely abundance of DNA fragments <200 bp in clinical samples (24). However, extracted genomic DNA is likely already significantly fragmented after initial nucleic acid extraction, and the units assigned to the corresponding samples reflect an amount (genome equivalents) that is equivalent to a genome calculated from an initial measured DNA concentration (i.e., ng/µL) and by assuming one genome per fungal cell provides a more representative indication of fungal burden compared to DNA concentration alone. For clinical compatibility, upcoming interlaboratory assays should focus on providing simulated cell-free DNA samples by using a short fragment of DNA (fragmentase-digested DNA), as previously done for investigation of preanalytical variables impacting the detection of other pathogen cell-free DNA in blood (24). A comparison of performance when using larger volume of serum or plasma (4–10 mL) would also be particularly interesting and helpful in initiating a shift toward routine tests based on larger sample volume.

Nevertheless, through the efforts of FPCRI M-LWP, various technical parameters influencing Mucorales qPCR performance (including six different automated DNA extraction platforms, eight different qPCR assays, and six amplification platforms) were evaluated. Using contrived sample from Panel A, positivity rates for the IH1 assay were superior to the commercial assays. The lower commercial sensitivity could be related to the different PCR targets, but is probably mainly due to the lower template percentage used in accordance with the supplier’s recommendations: 20% for MucorGenius and 40% for Fungiplex, compared with 45% for the in-house technique. Indeed, only sample volumes (initial volume of serum for extraction and volume of DNA extract used in the final PCR amplification) were significant parameters for optimal performance, as demonstrated for *Aspergillus* PCR some years ago (27). This is encouraging, as mycologists responsible for molecular diagnostics generally have limited equipment options, utilizing shared molecular platforms. Encouragingly, the preanalytical variables required to optimize fungal qPCR can be easily implemented, by simply performing DNA extraction using at least 1 mL plasma/serum sample volume coupled to the lowest possible elution volume (50 µL) and using the largest DNA input volume (7–10 µL) in the final PCR reaction (20–25 µL) and is irrespective of the extraction platforms and qPCR assay/platform. Our study also highlights the concern with the EU directives demanding the use of IVDR commercial diagnostics as they are not necessarily optimal, particularly when compared to well-established in-house methods and when a broad standardization of other technical parameters (sample volume, elution volume, PCR input volume, and template percentage) is pivotal for improved performance. Only a quality approach including external controls and interlaboratory tests can help to distinguish optimal tests, irrespective of technique, commercial, or in-house design. Accreditation is possible for both approaches and should be the only requirement for guaranteeing the quality of a test in a clinical setting.

## SUPPLEMENTAL MATERIAL

Supplemental methods jcm.01525-24-s0001.docx Additional data regarding qPCR methods.

Table S1 jcm.01525-24-s0002.docx Technical details of extraction methods.

Table S2 jcm.01525-24-s0003.docx Primers and probes for in-house technique.

Table S3 jcm.01525-24-s0004.docx Detection channel distribution of Mucorales PCR kit.

Table S4 jcm.01525-24-s0005.docx Technical details of qPCR methods.

Table S5 jcm.01525-24-s0006.docx qPCR assay sensitivity according to volume parameters.

ASM does not own the copyrights to Supplemental Material that may be linked to, or accessed through, an article. The authors have granted ASM a non-exclusive, world-wide license to publish the Supplemental Material files. Please contact the corresponding author directly for reuse.
