# Supplementary material for: Interlaboratory assays from the fungal PCR Initiative and the Modimucor Study Group to improve qPCR detection of Mucorales DNA in serum: one more step toward standardization
Source: J Clin Microbiol. 2024 Dec 31;63(2):e01525-24. doi: 10.1128/jcm.01525-24 (PMC11837492; doi:10.1128/jcm.01525-24)
Supplement: Supplemental methods — Additional data regarding qPCR methods. [file jcm.01525-24-s0001.docx]

***Supplementary Data***

***Methods***

***QPCR assays (Panel A)***

The Mucorales IH1 PCR assay technique combines one simplex assay which allows both the detection of *Mucor* and *Rhizopus* DNA (without genera distinction), and a second duplex assay which allows the detection of *Lichtheimia* DNA and *Rhizomucor* DNA separately. Each qPCR assay was performed in a final reaction volume of 20 µL using 1 µL of probe and primers pre-mix, 10 µL of TAKYON master-mix (Eurogentec, Brussels, Belgium) and 9 µL of DNA extract (supplementary Table 2). PCR amplification onto the Quantstudio 5, used the following program: 1 min at 50°C; 10 min at 95°C; 50 cycles with 15 sec at 95°C and 1 min at 60°C and 1 min at 40°C.

The MucorGenius kit targets the 18S rDNA region and has been design to detect simultaneously *Mucor* spp*, Rhizopus* spp*, Rhizomucor* spp, *Lichtheimia spp* and *Cunninghamella* spp, without genera distinction. The qPCR was performed using 5 μL of DNA extract in a final reaction volume of 25 μL containing 10 µL of MucorGenius PCR mix, 1 µL of Taq polymerase and 9 µL of dilution buffer following the supplier’s instructions on a QuantStudio 5 using the following program: 2 min at 95°C and 45 cycles with 15 sec at 94°C and 1 min at 58°C (Supplementary Table 3).

The Fungiplex Mucorales qPCR assay was performed using 5 µL of DNA extract in a final reaction volume of 20 µL containing 14 μL of master-mix (10 µL PCR Mix; 1.5 µL Mucorales mix; 2.5 µL PCR water) and 1 µL of an inhibition control provided in the kit. Detection of the inhibition control was done via the FAM channel and the nine different Mucorales genera were detected in any of the other three detection channels (Supplementary Table 3). The qPCR was performed according to the supplier's instructions on a Quantstudio using the following programme (2 min at 95°C; 45 cycles with 15 sec at 94°C and 1 min at 58°C) and thresholds were adjusted as recommended.
