# Supplementary material for: Interlaboratory assays from the fungal PCR Initiative and the Modimucor Study Group to improve qPCR detection of Mucorales DNA in serum: one more step toward standardization
Source: J Clin Microbiol. 2024 Dec 31;63(2):e01525-24. doi: 10.1128/jcm.01525-24 (PMC11837492; doi:10.1128/jcm.01525-24)
Supplement: Table S1 — Technical details of extraction methods. [file jcm.01525-24-s0002.docx]

**Supplementary Table 1**: Technical details of extraction methods

| **Centre** | **Sample volume used for DNA extraction (µL)** | **Elution volume (µL)** | **platform** | **DNA extraction kit** | **Code** |
| --- | --- | --- | --- | --- | --- |
|  |  |  |  |  |  |
| **C1** | 1000 | 85 | QIAsymphony (Qiagen) | Virus/ Pathogen Midi Kit | Q |
| **C2** | 1000 | 100 | ELITe Ingenius  (ELITech Group) | ELITe InGenius SP1000 | I |
| **C3** | 1000 | 165 | QIAsymphony (Qiagen) | QIAsymphony DSP DNA DNAmidi kit | Q |
| **C4** | 1000 | 50 | NucliSENS easyMAG (bioMérieux) | NucliSENS easyMAG Extraction Kit | B |
| **C5** | 1000 | 50 | NucliSENS easyMAG (bioMérieux)) | NucliSENS easyMAG Extraction Kit | B |
| **C6** | 1000 | 50 | NucliSENS easyMag (bioMérieux) | NucliSENS easyMAG Extraction Kit | B |
| **C7** | 1000 | 50 | NucliSENS easyMAG (bioMérieux) | NucliSENS easyMAG Extraction Kit | B |
| **C8** | 1000 | 50 | MagNA Pure 24 (Roche) | MagNA Pure 24 Total NA Isolation Kit | R |
| **C9** | 1000 | 50 | MagNA Pure 24 (Roche) | MagNA Pure 24 Total NA Isolation Kit | R |
| **C10** | 1000 | 50 | MagNA Pure 24 (Roche) | MagNA Pure 24 Total NA Isolation Kit | R |
| **C11** | 1000 | 100 | Hamilton Microlab STARlet | Nucleospin 96 DNA Plasma Kit (Macherey Nagel) | H |
| **C12** | 1000 | 100 | ELITech Ingenius  (ELITech group) | ELITe InGenius SP1000 | I |
| **C13** | 500 | 50 | MagNA Pure 96 (Roche) | MagNA Pure 96 DNA and Viral NA Large Volume Kit | R |
| **C14** | 1000 | 65 | None (Manual) | QIAamp UltraSens Virus Kit | M |
| **C15** | 200 | 100 | MT-Prep 24 (AusDiagnostics) | MT-Prep Viral/Pathogen Extraction Kit | A |
| **C16** | 500 | 75 | NucliSENS easyMAG  (bioMérieux) | NucliSENS easyMAG Extraction Kit | B |
| **C17** | 300 | 50 | None (Manual) | High Pure PCR Template Preparation Kit (Roche) | M |
| **C18** | 400 | 100 | MagNA Pure Compact (Roche) | Nucleic Acid Isolation Kit I - Large Volume | R |
| **C19** | 200 | 50 | None (Manual) | QIAamp DNA Blood Mini kit (Qiagen) | M |
| **C20** | 200 | 50 | None (Manual) | QIAamp DNA Blood Mini kit (Qiagen) | M |
| **C24** | 1000 | 50 | MagNA Pure Compact (Roche) | Nucleic Acid Isolation Kit I - Large Volume | R |
| **C26** | 1000 | 100 | NucliSENS easyMAG  (bioMérieux) | NucliSENS easyMAG Extraction Kit | B |
| **C27** | 200 | 100 | ELITe Ingenius  (ELITech Group) | ELITe InGenius SP200 | I |
| **C28** | 200 | 50 | None (Manual) | QIAmp DNA Mini Kit (Qiagen) | M |
| **C29** | 200 | 50 | MagNA Pure (Roche) | MagNA Pure 96 Total NA Isolation Kit | R |
| **C30** | 400 | 60 | EZ1 Advanced XL (Qiagen) | Automated Virus Pathogen EZ1 | Q |

Automated method: Qiagen (Q); Roche (R); Ingenius (I); bioMérieux (B); Hamilton (H); AusDiagnostics (A)

Manual methods (M)
