# Supplementary material for: Interlaboratory assays from the fungal PCR Initiative and the Modimucor Study Group to improve qPCR detection of Mucorales DNA in serum: one more step toward standardization
Source: J Clin Microbiol. 2024 Dec 31;63(2):e01525-24. doi: 10.1128/jcm.01525-24 (PMC11837492; doi:10.1128/jcm.01525-24)
Supplement: Table S2 — Primers and probes for in-house technique. [file jcm.01525-24-s0003.docx]

**Supplementary Table 2**: Primers and probes for in-house technique (IH1, described by Millon et al. 2016)

| **Target** | **Primer and Probe** | **Sequences** | **Type of qPCR** | **final concentrations of primers and probes** | **Channel** |
| --- | --- | --- | --- | --- | --- |
| *Mucor*/*Rhizopus* | Forward primer | CACCGCCCGTCGCTAC | Simplex | 1µM | FAM |
|  | Reverse primer : | CCTAGTTTGCCATAGTTCTCTGCAG |  | 1µM |  |
|  | Probe | FAM-CCGATTGAATGGTTATAGTGAGCATATGGGATC-TAMRA |  | 0.08µM |  |
| *Lichtheimia* | Forward primer | CACCGCCCGTCGCTAC * | Duplex | 1µM | FAM |
|  | Reverse primer | GCAAAGCGTTCCGAAGGACA |  | 1µM |  |
|  | Probe | FAM-ATGGCACGAGCAAGCATTAGGGACG-TAMRA |  | 0.08µM |  |
| *Rhizomucor* | Forward primer | CACCGCCCGTCGCTAC * |  | 1µM | VIC (HEX) |
|  | Reverse primer | GTAGTTTGCCATAGTTCGGCTA |  | 1µM |  |
|  | Probe | HEX-TTGAATGGCTATAGTGAGCATATGGGAGGCT-TAMRA |  | 0.1µM |  |

*Same forward primer was used for duplex amplification of *Lichtheimia* and *Rhizomucor*.
