# Supplementary material for: Interlaboratory assays from the fungal PCR Initiative and the Modimucor Study Group to improve qPCR detection of Mucorales DNA in serum: one more step toward standardization
Source: J Clin Microbiol. 2024 Dec 31;63(2):e01525-24. doi: 10.1128/jcm.01525-24 (PMC11837492; doi:10.1128/jcm.01525-24)
Supplement: Table S3 — Detection channel distribution of Mucorales PCR kit. [file jcm.01525-24-s0004.docx]

**Supplementary Table 3**: Detection channel distribution of Mucorales PCR kit (QuantStudio 5, ThermoFisher Scientific)

| **Kits** | **Channel 1 - FAM** | **Channel 2 – VIC/HEX** | **Channel 3 –**  **ROX** | **Channel 4 –**  **Cy5** |
| --- | --- | --- | --- | --- |
| Fungiplex^®^ Mucorales RUO PCR Kit (Bruker, Bremen, Germany) | Inhibition control | *Lichtheimia* spp.  *Syncephalastrum* spp. | *Rhizopus* spp. | *Rhizomucor* spp. |
|  |  |  | *Mucor* spp. |  |
|  |  |  | *Cuninghamella* spp. |  |
|  |  |  | *Actinomucor* spp. |  |
|  |  |  | *Apophysomyces* spp. |  |
|  |  |  | *Saksenaea* spp. |  |
| MucorGenius^®^ (PathoNostics, Maastricht, The Netherlands) | Mucorales spp. | Inhibition control |  |  |
