# Supplementary material for: Interlaboratory assays from the fungal PCR Initiative and the Modimucor Study Group to improve qPCR detection of Mucorales DNA in serum: one more step toward standardization
Source: J Clin Microbiol. 2024 Dec 31;63(2):e01525-24. doi: 10.1128/jcm.01525-24 (PMC11837492; doi:10.1128/jcm.01525-24)
Supplement: Table S4 — Technical details of qPCR methods. [file jcm.01525-24-s0005.docx]

**Supplementary Table 4**: Technical details of qPCR methods

| **Center number** | **Input qPCR volume (DNA in μl)** | **Final qPCR volume (μl)** | **qPCR assay** | **Code qPCR assay** | **qPCR platform** | **Code qPCR platform :** |
| --- | --- | --- | --- | --- | --- | --- |
| **C1** | 9 | 25 | In-house (2) | IH1 | LC480 II | R2 |
| **C2** | 9 | 20 | In-house (2) | IH1 | LC480 II | R2 |
| **C3** | 9 | 20 | In-house (2) | IH1 | Step One Plus | AB |
| **C4** | 9 | 25 | In-house (2) | IH1 | LC480 II | R2 |
| **C5** | 9 | 20 | In-house (2) | IH1 | LC480 | R2 |
| **C6** | 9 | 20 | In-house (2) | IH1 | QuantStudio5 | AB |
| **C7** | 5 | 20* | MucorGenius (PathoNostics) | MP  (modified) | QuantStudio 5 | AB |
| **C8** | 9 | 25 | In-house (2) | IH1 | LC480 | R2 |
| **C9** | 9 | 25 | In-house (2) | IH1 | RotorGene Q | Q |
| **C10** | 10 | 25 | In-house (2) | IH1 | QuantStudio 5 | AB |
| **C11** | 9 | 20 | In-house (2) | IH1 | Applied 7500 | AB |
| **C12** | 10 | 25 | MycoGENIE (Ademtech) | MA | InGenius (ELITech) | I |
| **C13** | 8 | 20 | In-house mitochondrial DNA (unpublished) | Other | LC480 II | R2 |
| **C14** | 5 | 20 | In-house (28) | IH3 | Step One Plus | AB |
| **C15** | 5 | 25 | In-house (19) | IH4 | RotorGene 6000 | Q |
| **C16** | 5 | 25 | MucorGenius (PathoNostics) | MP | RotorGene (Qiagen) | Q |
| **C17** | 5 | 20 | In-house (20) | IH5 | LightCycler 2.0 (Roche) | R1 |
| **C18** | 7 | 20 | In-house (2) | IH1 | CFX96 (Bio-Rad) | B |
| **C19** | 8 | 10 | In-house mitochondrial DNA  (unpublished) | Other | CFX96 (Bio-Rad) | B |
| **C20** | 1.5** | 25** | In-house (2) | IH1  (modified) | QuantStudio 5 | AB |
| **C24** | 5 | 20 | In-house (2) | IH1 | LC480 | R2 |
| **C26** | 5 | 30 | In-house (18) | IH2 | QuantStudio 5 | AB |
| **C27** | 6 | 15 | In-house 18S rDNA  (unpublished) | Other | InGenius (ELitech) | I |
| **C28** | 2 | 20 | In-house (21) | IH6 | LC480 II | R2 |
| **C29** | 5 | 50 | In-house (18) | IH2 | LC480 | R2 |
| **C30** | 5 | 25 | MucorGenius (PathoNostics) | MP | MIC | MIC |

* different from volume recommended by suppliers

** different from volume recommended by authors

Bernal-Martinez, L., M. J. Buitrago, M. V. Castelli, J. L. Rodriguez-Tudela, and M. Cuenca-Estrella. 2013. "Development of a single tube multiplex real-time PCR to detect the most clinically relevant Mucormycetes species." *Clin Microbiol Infect* 19 (1): E1-7. https://doi.org/10.1111/j.1469-0691.2012.03976.x.

Camp, I., G. Manhart, C. Schabereiter-Gurtner, K. Spettel, B. Selitsch, and B. Willinger. 2020. "Clinical evaluation of an in-house panfungal real-time PCR assay for the detection of fungal pathogens." *Infection* 48 (3): 345-355. https://doi.org/10.1007/s15010-020-01395-7. https://[www.ncbi.nlm.nih.gov/pubmed/32052286](http://www.ncbi.nlm.nih.gov/pubmed/32052286).

Lengerova, M., Z. Racil, K. Hrncirova, I. Kocmanova, P. Volfova, D. Ricna, P. Bejdak, M. Moulis, Z. Pavlovsky, B. Weinbergerova, M. Toskova, and J. Mayer. 2014. "Rapid detection and identification of mucormycetes in bronchoalveolar lavage samples from immunocompromised patients with pulmonary infiltrates by use of high-resolution melt analysis." *J Clin Microbiol* 52 (8): 2824-8. https://doi.org/10.1128/JCM.00637-14. https://[www.ncbi.nlm.nih.gov/pubmed/24850354](http://www.ncbi.nlm.nih.gov/pubmed/24850354).

Millon, L., R. Herbrecht, F. Grenouillet, F. Morio, A. Alanio, V. Letscher-Bru, S. Cassaing, T. Chouaki, C. Kauffmann-Lacroix, P. Poirier, D. Toubas, O. Augereau, S. Rocchi, D. Garcia-Hermoso, S. Bretagne, and French Mycosis Study Group. 2016. "Early diagnosis and monitoring of mucormycosis by detection of circulating DNA in serum: retrospective analysis of 44 cases collected through the French Surveillance Network of Invasive Fungal Infections (RESSIF)." *Clin Microbiol Infect* 22 (9): 810.e1-810.e8. https://doi.org/10.1016/j.cmi.2015.12.006. https://[www.ncbi.nlm.nih.gov/pubmed/26706615](http://www.ncbi.nlm.nih.gov/pubmed/26706615).

Salehi, E., M. T. Hedayati, J. Zoll, H. Rafati, M. Ghasemi, A. Doroudinia, M. Abastabar, A. Tolooe, E. Snelders, H. A. van der Lee, A. J. Rijs, P. E. Verweij, S. Seyedmousavi, and W. J. Melchers. 2016. "Discrimination of Aspergillosis, Mucormycosis, Fusariosis, and Scedosporiosis in Formalin-Fixed Paraffin-Embedded Tissue Specimens by Use of Multiple Real-Time Quantitative PCR Assays." *J Clin Microbiol* 54 (11): 2798-2803. https://doi.org/10.1128/JCM.01185-16. https://[www.ncbi.nlm.nih.gov/pubmed/27605714](http://www.ncbi.nlm.nih.gov/pubmed/27605714).

Springer, J., D. Goldenberger, F. Schmidt, M. Weisser, E. Wehrle-Wieland, H. Einsele, R. Frei, and J. Löffler. 2016. "Development and application of two independent real-time PCR assays to detect clinically relevant Mucorales species." *J Med Microbiol* 65 (3): 227-34. https://doi.org/10.1099/jmm.0.000218. https://[www.ncbi.nlm.nih.gov/pubmed/26743820](http://www.ncbi.nlm.nih.gov/pubmed/26743820).
