# Supplementary material for: Interlaboratory assays from the fungal PCR Initiative and the Modimucor Study Group to improve qPCR detection of Mucorales DNA in serum: one more step toward standardization
Source: J Clin Microbiol. 2024 Dec 31;63(2):e01525-24. doi: 10.1128/jcm.01525-24 (PMC11837492; doi:10.1128/jcm.01525-24)
Supplement: Table S5 — qPCR assay sensitivity according to volume parameters. [file jcm.01525-24-s0006.docx]

**Supplemental Table 5 :**  qPCR assay sensitivity according to volumes parameters for DNA extraction for IH1, Fungiplex and Mucorgenius

| Panels | | qPCR | | Volumes | | Sensitivity | | Fisher's Exact Test | |
| --- | --- | --- | --- | --- | --- | --- | --- | --- | --- |
| Panel A  DNA extraction | IH1 | | Sample volume = 1 mL | | 92.9% | | 0.11 | |  |
|  |  |  | Sample volume < 1 mL | | 83.3% | |  |  |  |
|  |  |  | Elution volume = 50 µL | | 91.0% | | 0.43 | |  |
|  |  |  | Elution volume > 50 µL | | 86.4% | |  |  |  |
|  | Fungiplex | | Sample volume = 1 mL | | 82.1% | | < 0.01 | |  |
|  |  |  | Sample volume < 1 mL | | 53.3% | |  |  |  |
|  |  |  | Elution volume = 50 µL | | 71.8% | | 0.72 | |  |
|  |  |  | Elution volume > 50 µL | | 68.2% | |  |  |  |
|  | Mucorgenius | | Sample volume = 1 mL | | 72.6% | | 0.01 | |  |
|  |  |  | Sample volume < 1 mL | | 51.7% | |  |  |  |
|  |  |  | Elution volume = 50 µL | | 66.7% | | 0.49 | |  |
|  |  |  | Elution volume > 50 µL | | 60.6% | |  |  |  |

Panel A: serum and elution volumes used in the DNA extraction step (24 centres, 144 qPCR trials by qPCR assays) (2 centres excluded from the analysis due to contamination or PCR inhibition).
